# Use of Diltiazem in Chronic Rate Control for Atrial Fibrillation: A Prospective Case-Control Study

**DOI:** 10.3390/biology12010022

**Published:** 2022-12-22

**Authors:** Igor Diemberger, Alberto Spadotto, Giulia Massaro, Martina Amadori, Liviu Damaschin, Cristian Martignani, Matteo Ziacchi, Mauro Biffi, Nazzareno Galiè, Giuseppe Boriani

**Affiliations:** 1 Department of Experimental, Diagnostic and Specialty Medicine, Alma Mater Studiorum—University of Bologna, 40138 Bologna, Italy; 2 IRCCS Policlinico S.Orsola-Malpighi, U.O.C. di Cardiologia, 40138 Bologna, Italy; 3 Cardiology Division, Department of Biomedical, Metabolic and Neural Sciences, University of Modena and Reggio Emilia, Policlinico di Modena, 41125 Modena, Italy

**Keywords:** personalised therapy, calcium channel blockers, beta-blockers, survival, mortality

## Abstract

**Simple Summary:**

Recent ESC Atrial Fibrillation guidelines introduced some changes in the options for rate control, such as the possibility to combine beta-blockers and non-dihydropyridine calcium channel blockers to address the need for a personalised pharmacologic rate control treatment for AF. However, there are limited data on this topic. This real-world prospective observational study aims to explore the prognostic impact of a patient-specific therapy for rate control in atrial fibrillation, including the use of non-dihydropyridine calcium channel blockers in patients with heart failure or in combination with beta-blockers, compared to standard rate control therapy, as defined by previous ESC guidelines. We performed an analysis of 1112 patients on exclusive rate control treatment referred to our University Hospital. Our results showed no difference in the one-year overall survival in the patient-specific therapy group compared to the standard treatment group. The use of non-dihydropyridine calcium channel blockers for rate control in patients with atrial fibrillation, either alone or in combination with beta-blockers, showed clinical benefit in selected patients, including a group of subjects with heart failure. Future controlled studies are needed to confirm our findings and identify subjects who will obtain greater benefit from such patient-specific rate-control strategies.

**Abstract:**

Atrial fibrillation (AF) is a multifaceted disease requiring personalised treatment. The aim of our study was to explore the prognostic impact of a patient-specific therapy (PT) for rate control, including the use of non-dihydropyridine calcium channel blockers (NDDC) in patients with heart failure (HF) or in combination with beta-blockers (BB), compared to standard rate control therapy (ST), as defined by previous ESC guidelines. This is a single-centre prospective observational registry on AF patients who were followed by our University Hospital. We included 1112 patients on an exclusive rate control treatment. The PT group consisted of 125 (11.2%) patients, 93/125 (74.4%) of whom were prescribed BB + NDCC (±digoxin), while 85/125 (68.0%) were HF patients who were prescribed NDCC, which was diltiazem in all cases. The patients treated with a PT showed no difference in one-year overall survival compared to those with an ST. Notably, the patients with HF in ST had a worse prognosis (*p* < 0.001). To better define this finding, we performed three sensitivity analyses by matching each patient in the PT subgroups with three subjects from the ST cohort, showing an improved one-year survival of the HF patients treated with PT (*p* = 0.039). Our results suggest a potential outcome benefit of NDCC for rate control in AF patients, either alone or in combination with BB and in selected patients with HF.

## 1. Introduction

Atrial fibrillation (AF) is the most common sustained arrhythmia that has shown a progressive increase in prevalence due to population aging but also due to improved survival to acute illness [1]. AF, more than other rhythm disturbances, is a multifaceted clinical condition requiring personalisation of its management, as also stressed by the current guidelines [2]. According to the ABC approach, after assessing the need for anticoagulation, we have to identify the best approach for controlling the symptoms, either rhythm control or rate control. Despite recent evidence supporting rhythm-control strategies, in a specific subset of patients [3,4], rate control is still mandatory [5]. The last ESC guidelines introduced some changes in the options for rate control, such as the possibility to combine beta-blockers (BB) and non-dihydropyridine calcium channel blockers (NDCC) [2]. However, despite the absence of specific evidence hampering these options, there is limited data supporting them. We aimed our analysis at exploring the prognostic impact of a patient-specific therapy for rate control, including the use of NDDC in selected patients with heart failure (HF), or reduced left ventricular ejection fraction (LVEF), or in combination with BB, compared to standard rate control therapy, as defined by previous ESC guidelines [6,7].

## 2. Materials and Methods

### 2.1. Study Design and Patient Information

This is a single-centre prospective observational registry on patients referred to a University Hospital for the management of atrial fibrillation. The study design was previously described [8]. In brief, all the consecutive patients affected by AF admitted to, or followed up by, the Cardiology Department of the Sant’Orsola-Malpighi University Hospital in the period between October 2013 and February 2019 were enrolled. The criteria for enrolment were an age >18 years and at least one documented episode of AF in the 12 months prior to the hospitalisation or visit. AF did not necessarily have to be present at the time of inclusion and it did not necessarily have to be the reason for admission. The patients who presented with atrial flutter were included only in the case of at least one ECG documented episode of AF. 

The present analysis was aimed at exploring the use of a “patient-specific therapy” (PT) for rate control, including the use of NDDC in patients with HF and/or in combination with BB, compared to “standard therapy” (ST), according to previous ESC guidelines [6,7]. In particular, the “patient-specific therapy” for rate control was defined as one of the following regimens:

- BB + NDCC: for patients with and without HF
- BB + NDCC + digoxin: for patients with and without HF
- NDCC: for patients with HF
- NDCC + digoxin: for patients with HF

In the definition of HF, we included the following subgroups: (a) HFrEF: presenting both a left ventricular ejection fraction (LVEF) ≤ 35% and New York Heart Association (NYHA) class ≥ II symptoms; (b) HF-not-rEF: combining patients with heart failure with a preserved or mildly reduced ejection fraction (with an LVEF > 35% and NYHA class ≥ II symptoms) or asymptomatic left ventricle systolic dysfunction (ALVSD; with an asymptomatic LVEF ≤ 35%) [9].

### 2.2. Data Collection 

After the collection of informed consent, the pharmacological and instrumental data of the patients were obtained. The choice of therapy was made according to the clinical practice of the centre, regardless of study participation. 

All the patients were followed for at least 12 months. All the enrolled patients underwent a one-year follow-up evaluation carried out during an outpatient cardiological visit. Interim evaluations were conducted according to clinical practice or need. In the case of death, the date and reason were indicated. The primary end point was one-year mortality. 

The study was performed in accordance with the principles of the Declaration of Helsinki and was approved by the Local Ethics Committee. Written informed consent was signed by every patient before enrolment. 

### 2.3. Statistical Analysis

The continuous variables are expressed as the mean ± standard deviation, if normally distributed; otherwise, they are expressed as the median and interquartile ranges. The discrete variables are expressed as frequencies and percentages. Comparisons for continuous data were performed via Student’s *t*-tests, and Chi2 testing was applied to the categorical data. Kaplan–Meier survival curves estimated the unadjusted survival distributions from death. 

In order to obviate possible selection bias derived from a non-randomised design, we performed three sensitivity analyses, matching each patient in the PT cohort with subjects from the ST cohort who were randomly chosen based on the following covariates sorted in order: age, sex, LVEF and digoxin intake, in view of their known effect on the clinical outcomes and choice of treatment. The sensitivity analyses were performed on three subgroups chosen according to the criteria used to define the patient-specific therapy: the first group was composed of patients taking BB + NDCC (±digoxin), the second was composed of patients with HF taking only NDCC (±digoxin) for rate control therapy and the third was composed of all the patients with HF in the PT group. Given the number of patients in the PT and ST groups, a 1:3 matching ratio was chosen, and the matching was conducted without replacement. The matched analysis provides an intuitive presentation of the patient characteristics in “comparable” exposure groups matched on important confounding factors; however, residual confounding remains a possibility given the difficulties in accounting for all the potential confounders [10,11,12].

We used SPSS 23.0 (SPSS Statistics/IBM Corp, Chicago IL, USA) for statistical analysis, and *p* values < 0.05 were considered significant.

## 3. Results

### 3.1. Clinical Characteristics

The overall cohort included 1435 patients and 1112 patients on an exclusive rate control treatment were included in the analysis. The patients with paroxysmal AF in whom a rhythm control or wait-and-see approach was attempted were excluded, whereas the patients with a first AF episode who were treated with an exclusive rate control were included. Of the patients on an exclusive rate control treatment, 125 (11.2%) were identified as patients with PT and 987 (88.8%) were on ST, according to the aforementioned criteria. The baseline clinical data of the ST and PT groups are summarised in Table 1. The mean ages of the patients were 73.5 ± 12.0 years and 72.5 ± 12.7 years in the ST and PT groups, respectively. The two cohorts were homogeneous in terms of male prevalence (62.8% in ST vs. 62.4% in PT) and the LVEF (53.3% ± 15.0% in ST vs. 53.0% ± 15.2% in PT). Heart failure was more frequent in the PT group in terms of the patients with a NYHA class ≥ 2 (41.3% in ST vs. 64.8% in PT, *p* < 0.001), the prescription of diuretics (72.2% in ST vs. 88.0% in PT, *p* < 0.001) and digoxin (9.9% in ST vs. 17.6% in PT, *p* = 0.009). Moreover, the PT group showed a greater proportion of patients with a prior ischemic stroke (4.9% in ST vs. 11.2% in PT, *p* = 0.004) or previous thromboembolic events (4.1% in ST vs. 8.8% in PT, *p* = 0.017). The risk of thromboembolic events and bleeding, according to the CHA2DS2-VASc and HAS-BLED score, was comparable between the two groups. The use of anticoagulant drugs differed between the ST and PT groups. In the latter, the use of warfarin was more frequent (70.3% in ST vs. 82.4% in PT, *p* = 0.005), while DOAC users were not different (13,0% in ST vs. 11.3% in PT). The use of NDCC significantly differed between the two groups in view of their defining criteria (5.3% in ST vs. 100% in PT, *p* < 0.001) and, in particular, in all the patients, the chosen NDCC was diltiazem. The number of patients under BB was similar in both groups (70.8% in ST vs. 74.4% in ST).

The subgroup of patients taking BB + NDCC (±digoxin) (Sensitivity analysis 1) was composed of 93 patients, the subgroup of patients with HF taking NDCC (±digoxin) (Sensitivity analysis 2) was composed of 32 patients and the subgroup of all the patients with HF in the PT group (Sensitivity analysis 3) was composed of 85 patients. The three subgroups of patients in PT therapy are described in Table 2.

#### 3.1.1. Subgroup BB + NDCC (±Digoxin) (Sensitivity Analysis 1)

The 93 patients of the PT group taking BB + NDCC (±digoxin) were matched at a ratio of 1:3 (see above), with a control group of 279 patients derived from the ST group (ST control group 1). The matched variables included age, sex, LVEF and digoxin intake. The baseline characteristics of the patients taking BB + NDCC (±digoxin) and ST control group 1 are summarised in Table 3. The patients taking BB + NDCC (±digoxin) had a comparable ischemic and bleeding risk to that of the control group; however, in the the BB + NDCC (±digoxin) subgroup a greater proportion of patients had a prior ischemic stroke (11.8% vs. 3.9% in ST control group 1, *p* = 0.005). In the subgroup taking BB + NDCC (±digoxin), there were more patients with a NYHA class ≥ 2 (54.8% vs. 39.8% in ST control group 1, *p* = 0.011) and a greater use of diuretics (84.9% vs. 74.2% in ST control group 1, *p* = 0.033). The use of warfarin was more frequent in the patients taking BB + NDCC (±digoxin) (82.8% vs. 71.0% in ST control group 1, *p* = 0.024), while the DOAC users did not differ (12.9% vs. 12.3% in ST control group 1).

#### 3.1.2. Subgroup NDCC (±Digoxin) in HF (Sensitivity Analysis 2)

The 32 patients of the PT group with HF taking NDCC (±digoxin) for rate control therapy were matched at a ratio of 1:3 with 96 patients with HF belonging to the ST group. This control group was named ST control group 2. The baseline characteristics are summarised in Table 3. The baseline characteristics of the patients with HF taking NDCC (±digoxin) and ST control group 2 were comparable, except for a higher percentage of patients with coronary artery disease in ST control group 2 (12.5% vs. 36.5% in ST control group 2, *p* = 0.001).

#### 3.1.3. Subgroup HF in PT (Sensitivity Analysis 3)

In the PT group, there were 85 patients with HF according to the aforementioned criteria. These patients were matched at a ratio of 1:3 with 255 patients with HF belonging to the ST group (ST control group 3). The baseline characteristics of these two populations are summarised in Table 3. The two subgroups were homogeneous in terms of the LVEF (49.1% ± 16.0% vs. 47.3% ± 16.6% in ST control group 3) and diuretics use (95.3% vs. 92.9% in ST control group 3). The patients with HF in PT were more symptomatic, with a higher percentage of NYHA class ≥ 2, although this difference was not statistically significant (95.3% vs. 88.6% in ST control group 3, *p* = 0.072). The patients with HF in PT group had a comparable ischemic and bleeding risk to that of the ST controls but had a greater number of prior ischemic strokes (11.8% vs. 3.1% in ST control group 3, *p* < 0.001). In view of the criteria used to create the subgroup, NDCC were used only in the PT subgroup. The use of BB was more frequent in ST control group 3 (62.4% vs. 76.9% in ST control group 3, *p* = 0.009).

### 3.2. One-Year All-Cause Survival

**Figure 1:**
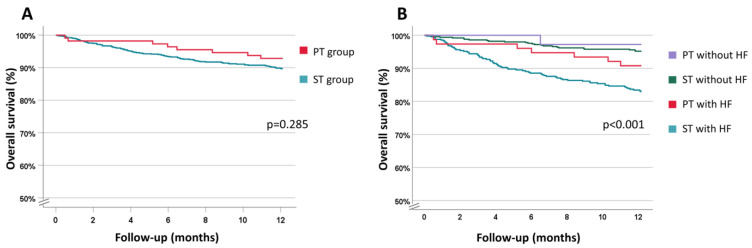
Kaplan–Meier curve. (**A**) Overall survival in the ST vs. PT groups. (**B**) Overall survival in the ST and PT groups stratified according to the presence of HF. PT (patient-specific therapy), ST (standard therapy).

At one year, 91 patients (8.2%) were lost at follow-up, with no difference between the two groups (8.1% in the ST group vs. 8.8% in the PT group, *p* = 0.788). At follow-up, there were 102 deaths (9.2%), 94 in the ST group (9.5%) and 8 in the PT group (6.4%). A comparison of the Kaplan–Meier survival curves showed a difference in the overall survival at one year (Figure 1A).

In stratifying the ST and PT groups according to the presence of HF, it emerged that the patients with HF who were treated with ST had a worse prognosis compared to the other subgroups (Figure 1B). To better define these results and avoid selection bias, a sensitivity analysis was conducted. The survival analyses between the three aforementioned PT subgroups and their ST control groups were performed.

#### 3.2.1. Subgroup BB + NDCC (±Digoxin) (Sensitivity Analysis 1)

**Figure 2:**
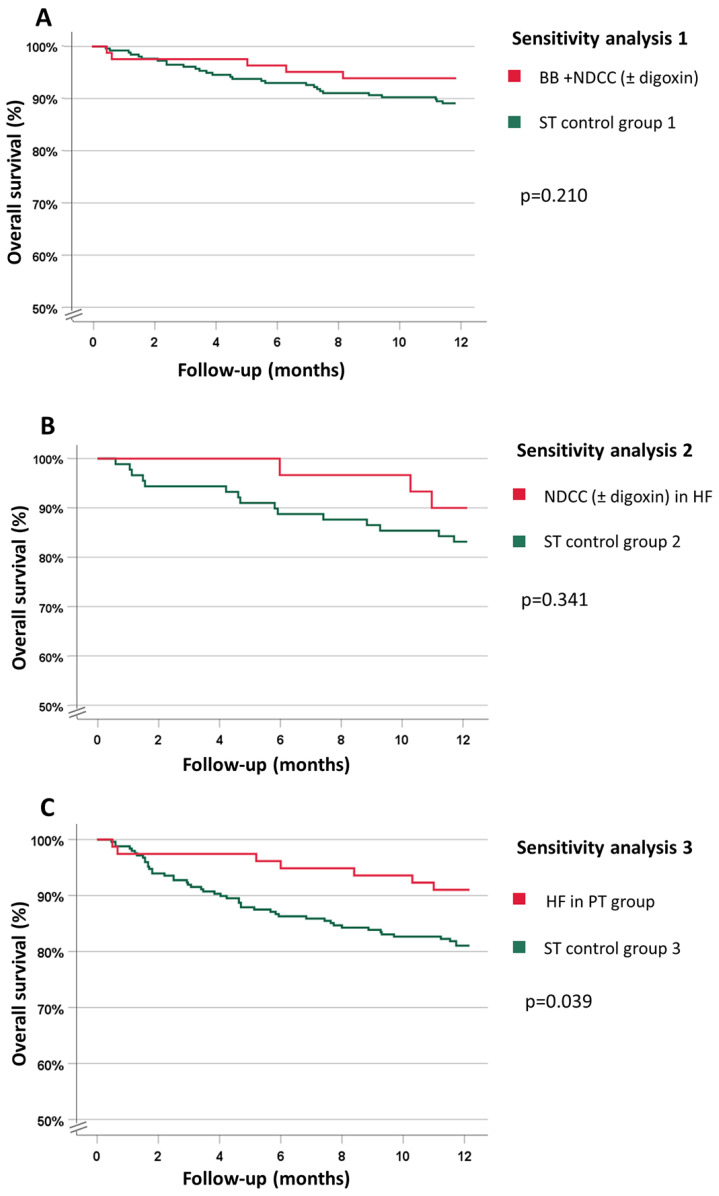
Kaplan–Meier curves. (**A**) Overall survival in patients prescribed BB + NDCC (digoxin) vs. ST Control group 1 (Sensitivity analysis 1); (**B**) Overall survival in patients with HF prescribed NDCC (±digoxin) vs. ST Control group 2 (Sensitivity analysis 2); (**C**) Overall survival in patients with HF in PT vs. ST Control group 3 (Sensitivity analysis 3). BB (beta-blockers), NDCC (non-dihydropyridine calcium channel blockers), PT (patient-specific therapy), ST (standard therapy).

At one year, there were 33 deaths (9.7%), 5 (6.1%) in the patients taking BB + NDCC (±digoxin) and 28 (10.9%) deaths in ST control group 1. A comparison of the Kaplan–Meier survival curves showed no difference in the one-year overall survival between the two groups (Figure 2A). 

#### 3.2.2. Subgroup NDCC (±Digoxin) in HF (Sensitivity Analysis 2)

At the end of the one-year follow-up, in the PT patients with HF taking NDCC (±digoxin), there were 3 deaths (10.0%) compared to 15 (16.9%) deaths in ST control group 2. The patients with HF taking NDCC (±digoxin) presented a better survival trend compared to ST control group 2; however, this difference was not significant (Figure 2B).

#### 3.2.3. Subgroup HF in PT (Sensitivity Analysis 3)

At one year, there were 54 deaths (15.8%), 7 (8.2%) in the HF patients taking PT and 47 (18.4%) in ST control group 3. The Kaplan–Meier survival curves showed a difference in the one-year overall survival between the two subgroups (*p* = 0.039) (Figure 2C). 

## 4. Discussion

AF is a multifaceted disease requiring personalised treatment [2]. In view of the limited data available from the literature comparing the use of BB, NDCC or digoxin for rate control in patients with AF [13,14], or their combination, the previous ESC guidelines limited the options for rate control and mainly focused on the use of BB (with/without digoxin) considering NDCC (with/without digoxin) only for patients with a preserved LVEF [2,6,7]. In our cohort, about 11.2% of the patients were treated with a patient-specific therapy, including BB and NDCC in association and/or the use of NDCC in patients with HF, without any negative impact on the one-year survival. This is in line with the consideration that data suggesting a contraindication of NDCC in HF with a reduced LVEF are outdated, and the related studies were not primarily designed to address the efficacy and safety of rate control of AF in patients with HF [15]. In this regard, it is noteworthy that we found signals of the possible positive effects of a patient-specific therapy, including NDCC in selected patients. 

In our cohort, a therapy combining BB and NDCC was administered to 8.3% of the patients (including 0.9% of the patients taking a triple rate control therapy). Patients taking BB + NDCC (±digoxin) showed no difference in the one-year overall survival compared to the control group of patients taking the standard rate control therapy. This was despite the patients in the BB + NDDCC group showing a higher risk profile at the baseline (greater proportion of patients with a prior ischemic stroke, more patients with a NYHA class ≥ 2) [16,17] compared to the control group. Notably, previous trials such as the Atrial Fibrillation Follow-Up Investigation of Rhythm Management (AFFIRM) Study [13,18] and the Rate Control Efficacy in Permanent Atrial Fibrillation: a Comparison between Lenient versus Strict Rate Control II (RACE II) trial [19] reported a combination of BB and NDCC in 7% and 2.9% of patients, respectively. Previous studies on the association of BB and NDCC for the treatment of chronic coronary syndrome have raised some concerns about the development of severe bradycardia or atrioventricular block. The adverse events reported were uncommon but not negligible [20,21]. In a small study on the use of a combination therapy of BB and NDCC for the acute rate control of AF or flutter with a rapid rate response, 3.7% of the patients developed bradycardia, but only 0.7% developed symptomatic bradycardia requiring drug administration [22]. Unfortunately, no data on the restoration of the sinus rhythm or the type of AF (paroxysmal vs. persistent) were available. Notably, 11.8% of the patients were already taking a combination therapy of NDCC + BB before admission [22]. Further studies are needed to confirm the safety of such a combination therapy in a specific subset of patients (e.g., patients with paroxysmal vs. persistent/permanent AF) and to determine the best practice criteria for monitoring patients under combination therapy (e.g., prolonged vs. repeated heart rhythm monitoring). The higher proportion of HF patients in the PT subgroup taking BB + NDCC may be related to the difficulty in achieving adequate rate control without combination therapy in some subsets of patients, such as those with HF, in which a stricter rate control may be desirable [2,23,24,25].

The use of calcium antagonists is discouraged in patients with HF and reduced ejection fraction (HFrEF) due to the reported negative inotropic effects, albeit it should be considered the lesser effect of diltiazem when compared to verapamil [23]. Considering the high co-existence of AF and HF, this limitation (without any differentiation between different agents) represents a constraint in therapeutic management [25]. In our cohort, 7.6% of the patients were taking NDCC (diltiazem for all the patients) despite having HF, 2.9% of the patients were taking only NDCC (±digoxin) and 4.7% were taking BB + NDCC (±digoxin). Interestingly, while in the overall population the one-year survival did not differ between the two treatment subgroups, the patients with HF who were receiving ST presented an increased risk of death. In order to obviate to a possible selection bias (e.g., the patients with PT tended to have more HF symptoms with respect to the LVEF impairment) derived from the non-randomised design of our study, we performed three sensitivity analyses by matching patients according to the presence of HF and the type of treatment they received. These additional analyses evidenced that the patients with HF treated with PT showed better one-year survival. Looking at the literature, we can find small studies showing the safety and efficacy of diltiazem in the acute rate control of AF or flutter with moderate to severe HF [24,26,27]. However, the data on the efficacy and safety of the long-term use of NDCC in patients with AF and an ejection fraction < 40% are lacking [15]. This is mainly due to the habit of avoiding NDCC in patients with HF. This contraindication stems from studies on HFrEF patients and ischemic heart disease. In the Multicenter Diltiazem Postinfarction Trial [28], which compared diltiazem to a placebo in the setting of ischemic heart disease, patients with pulmonary congestion on a chest X-ray who were treated with diltiazem had a significant increase in cardiac mortality or recurrent nonfatal infarction compared to those who received a placebo. In a subsequent analysis, diltiazem was found to increase late-onset HF in patients with a LVEF ≤ 40% (21% vs. 12% for the placebo), while, it had no adverse effects in patients with an LVEF above 40 percent. Most of the patients (55%) were receiving concomitant beta-blockers [29]. Similar results were suggested in a retrospective analysis of the Studies of Left Ventricular Dysfunction (SOLVD) [30], while this was not confirmed in a retrospective analysis from the Survival and Ventricular Enlargement (SAVE) Trial [31]. Interestingly, a randomised trial including 186 patients with idiopathic dilated cardiomyopathy (LVEF < 50%) and NYHA class II or III found an improvement in the cardiac index (at rest and with exercise), the stroke volume index and the exercise capacity in patients treated with diltiazem vs. the placebo arm [32]. None of these studies focused on the use of NDCC for rate control in patients with co-existing AF. In our study, in the subgroup of patients with HF undergoing PT, there was a lower prevalence of ischemic heart disease compared to the control group, albeit the result was not statistically significant (31.8% vs. 40.8% in the control group, *p* = n.s.), and only a minority of patients with CAD were not prescribed concomitant BB (4/27 patients in the PT group). Considering the enrolment period, the use of angiotensin receptor neprilysin inhibitor (ARNI) and Sodium-glucose Cotransporter-2 (SGLT2) inhibitors was not evaluated. The NDCC anti-hypertensive effect could reduce the possibility of introducing these new drugs into therapy. In the PT group, where the use of NDCC was more extensive, the systolic blood pressure was lower than in the ST group (121.1 ± 16.3 vs. 125.4 ± 18.0, *p* = 0.012) however, in the subgroup HF patients with PT, there was no significant difference in the blood systolic pressure or in the use of ACE inhibitors/sartans compared to the ST controls.

The current ESC guidelines [2], despite recognising the limitations of the available literature, still support BB as the first choice for rate controls in AF patients, especially in subjects with HF. However, the benefits of BB on all-cause mortality in patients with AF and HF have been questioned by a recent metanalysis and re-evaluation of the literature [33,34,35,36]. Our findings support the changes proposed by these guidelines compared to previous recommendations, such as the possibility of associating BB and NDCC, and advocate for new studies on more extended use of NDCC for rate control in AF.

## 5. Limitations

This study is a monocentric observational study, and this poses inherent limitations by virtue of the study design. To mitigate the associated bias, we performed three sensitivity analyses. A total of 8.2% of the patients were lost at follow-up, introducing a potential bias, although there were no differences between the PT and ST groups. As this is a real-world observational study performed in a Tertiary University Hospital, residual confounding remains a possibility given the difficulties in accounting for all the potential confounders.

## 6. Conclusions

In summary, despite recent evidence on the positive effects of rhythm control in a specific subset of patients, rate control is still a pivotal element in the management of AF. The ESC AF guidelines have evolved over the years in an attempt to improve the personalisation of AF treatment, since AF is a multifaceted disease. Our results highlight the need for further personalisation in rate-control strategies regarding the role of NDCC and, in particular, diltiazem in selected patients, either alone or in combination with BB. However, new randomised studies are needed to confirm our findings and better define the subset of patients who can benefit most from these strategies.

## Figures and Tables

**Table 1:** Baseline characteristics of the standard therapy and patient-specific therapy groups.

	Standard Therapy (987 Patients)	Patient-Specific Therapy (125 Patients)	*p*-Value
**Age (years)**	73.5 ± 12.0	72.5 ± 12.7	0.366
**Male**	62.8%	62.4%	0.928
**BMI (kg/m^2^)**	26.5 ± 4.7	26.6 ± 4.9	0.794
**Systolic blood pressure (mmHg)**	125.4 ± 18.0	121.1 ± 16.3	**0.012**
**Hypertension**	73.2%	72.0%	0.771
**Diabetes**	21.7%	28.0%	0.110
**COPD**	17.6	21.6	0.28
**TIA**	5.8%	4.0%	0.411
**Ischemic stroke**	4.9%	11.2%	**0.004**
**Thromboembolic events**	4.1%	8.8%	**0.017**
**Major bleedings**	5.6%	5.6%	1.00
**Haemorrhagic stroke**	1.6%	2.4%	0.464
**Creatinine (mg/dL)**	1.2 ± 0.7	1.2 ± 0.5	0.532
**Ejection fraction (%)**	53.3 ± 15.0	53.0 ± 15.2	0.795
**NYHA class ≥ 2**	41.3%	64.8%	**<0.001**
**CAD**	38.5%	35.2%	0.477
**CHA_2_DS_2_-VASc score**	3.9 ± 1.8	4.1 ± 2.0	0.161
**CHA_2_DS_2_-VASc score ≥ 4**	60.4%	58.1%	0.613
**HAS-BLED score**	2.0 ± 0.9	2.0 ± 1.0	0.351
**EHRA score**	1.8 ± 1.5	2.0 ± 1.4	0.348
**Coumadin**	70.3%	82.4%	**0.005**
**DOAC**	13%	11.3%	0.575
**Aspirin**	29.3%	25.6%	0.390
**ACE inhibitors or Sartans**	56.5%	48.8%	0.104
**BB**	70.8%	74.4%	0.399
**Digoxin**	9.9%	17.6%	**0.009**
**Diuretics**	72.2%	88.0%	**<0.001**
**Aldosterone antagonists**	38.0%	46.3%	0.073
**NDCC**	5.3%	100.0%	**<0.001**

ACE (angiotensin-converting enzyme), BB (beta-blockers), BMI (body mass index), CAD (coronary artery disease), COPD (chronic obstructive pulmonary disease), DOAC (direct oral anticoagulant), NDCC (non-dihydropyridine calcium channel blockers), NYHA (New York Heart Association), TIA (transient ischemic attack).

**Table 2:**
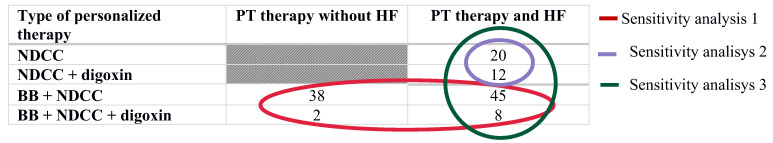
Patients in the PT group sorted according to therapeutic regimens. BB (beta-blockers), HF (heart failure), NDCC (non-dihydropyridine calcium channel blockers), PT (patient-specific therapy).

**Table 3:** Baseline characteristics of the patients in the PT subgroups and respective ST control groups.

	Sensitivity Analysis 1			Sensitivity Analysis 2			Sensitivity Analysis 3		
	BB + NDCC ±(Digoxin) (n° 93)	ST Control Group 1 (n° 79)	*p*-Value	NDCC (±Digoxin) in HF (n° 32)	ST Control Group 2 (n° 96)	*p*-Value	HF in PT Group(n° 85)	ST Control Group 3 (n° 255)	*p*-Value
Age (years)	72.8 ± 11.5	72.8 ± 11.7	1.000	71.8 ± 16.2	72.2 ± 12.9	0.877	72.0 ± 13.6	71.9 ± 12.0	0.978
Male	64.5%	64.5%	1.00	56.3%	56.3%	1.00	58.8%	62.4%	0.563
BMI (kg/m^2^)	27.1 ± 4.8	26.3 ± 4.4	0.109	25 ± 4.9	26.3 ± 5.0	0.224	26.8 ± 5.4	26.4 ± 4.7	0.444
Systolic blood pressure (mmHg)	122.0 ± 16.3	125.3 ± 18.2	0.119	118.6 ± 16.5	122.3 ± 19.3	0.334	118 ± 16.0	122 ± 19.5	0.128
Hypertension	72.0%	70.6%	0.792	71.9%	69.8%	0.823	69.4%	70.2%	0.891
Diabetes	29.0%	23.3%	0.267	25%	25%	1.00	31.8%	29.4%	0.682
COPD	19.4	18.3	0.82	28.1%	24.0%	0.637	25.9%	23.5%	0.661
TIA	4.3%	4.3%	1.00	3.1%	8.3%	0.318	3.5%	5.1%	0.554
Ischemic stroke	11.8%	3.9%	**0.005**	9.4	3.1	0.147	11.8%	3.1%	**<0.001**
Thromboembolic events	9.7%	4.3%	0.052	6.3	5.2%	0.822	9.4%	5.9%	0.262
Major bleedings	5.1%	4.8%	1.00	6.9%	7.5%	0.915	6.7%	5.8%	0.779
Haemorrhagic stroke	0%	1.8%	0.337	9.4	3.1	0.147	3.5%	2.4%	0.558
Creatinine (mg/dL)	1.2 ± 0.7	1.1 ± 0.5	0.074	1.27 ± 0.5	1.22 ± 0.5	0.627	1.2 ± 0.5	1.3 ± 0.8	0.171
Ejection fraction (%)	52.3 ± 15.5	52.4 ± 15.2	0.917	55.1 ± 14.4	53.1 ± 14.9	0.506	49.1 ± 16.0	47.3 ± 16.6	0.381
NYHA ≥ 2	54.8%	39.8%	**0.011**	93.8%	93.8%	1.00	95.3%	88.6%	0.072
CAD	43.0%	41.9%	0.856	12.5%	36.5%	**0.001**	31.8%	40.8%	0.139
CHA_2_DS_2_-VASc score	4.1 ± 2.0	3.7 ± 1.8	0.114	4.3 ± 2.0	4.4 ± 1.7	0.665	4.6 ± 2.0	4.3 ± 1.8	0.354
CHA_2_DS_2_-VASc score ≥4	57.0%	55.4%	0.794	65.6%	75.0%	0.303	65.5%	69.8%	0.458
HAS-BLED score	2.0 ± 0.9	2.0 ± 0.9	0.819	2.2 ± 1.0	2.0 ± 0.8	0.545	2.1 ± 1.0	2.1 ± 0.9	0.914
EHRA score	1.9 ± 1.6	2.1 ± 1.5	0.824	1.6 ± 1.2	1.8 ± 1.5	0.576	1.9 ± 1.5	1.8 ± 1.5	0.633
Coumadin	82.8%	71.0%	**0.024**	81.3%	87.5%	0.378	78.8%	85.1%	0.177
DOAC	12.9%	12.3%	0.835	9.3%	5.2%	0.500	15.3%	9.8%	0.250
Aspirin	29.0%	31.2%	0.697	15.6%	25%	0.273	18.8%	25.5%	0.211
ACE inhibitors or Sartans	49.5%	60.9%	0.052	46.9%	49.05	0.838	49.4%	53.3%	0.531
BB	100%	74.2%	**<0.001**	0%	71.9%	**<0.001**	62.4%	76.9%	**0.009**
Digoxin	10.8%	10.8%	1.00	37.5%	37.5%	1.00	23.5%	23.1%	0.941
Diuretics	84.9%	74.2%	**0.033**	96.9%	90.6%	0.254	95.3%	92.9%	0.445
Aldosterone antagonists	47.3%	39.1%	0.168	43.8%	50%	0.543	48.8%	55.3%	0.301
NDCC	100%	5.4%	**<0.001**	100%	0%	**<0.001**	100.0%	0%	**<0.001**

ACE (angiotensin-converting enzyme), BB (beta-blockers), BMI (body mass index), CAD (coronary artery disease), COPD (chronic obstructive pulmonary disease), DOAC (direct oral anticoagulant), HF (heart failure), NDCC (non-dihydropyridine calcium channel blockers), NYHA (New York Heart Association), TIA (transient ischemic attack).

## Data Availability

Due to privacy and ethical restrictions, the data are available by request only from the corresponding author.

## References

[1] Krijthe B.P., Kunst A., Benjamin E.J., Lip G.Y.H., Franco O.H., Hofman A., Witteman J.C.M., Stricker B.H., Heeringa J. (2013). Projections on the number of individuals with atrial fibrillation in the European Union, from 2000 to 2060. Eur. Heart J..

[2] Hindricks G., Potpara T., Dagres N., Arbelo E., Bax J.J., Blomström-Lundqvist C., Boriani G., Castella M., Dan G.-A., Dilaveris P.E. (2021). 2020 ESC Guidelines for the diagnosis and management of atrial fibrillation developed in collaboration with the European Association for Cardio-Thoracic Surgery (EACTS). Eur. Heart J..

[3] Marrouche N.F., Brachmann J., Andresen D., Siebels J., Boersma L., Jordaens L., Merkely B., Pokushalov E., Sanders P., Proff J. (2018). Catheter Ablation for Atrial Fibrillation with Heart Failure. N. Engl. J. Med..

[4] Kirchhof P., Camm A.J., Goette A., Brandes A., Eckardt L., Elvan A., Fetsch T., van Gelder I.C., Haase D., Haegeli L.M. (2020). Early Rhythm-Control Therapy in Patients with Atrial Fibrillation. N. Engl. J. Med..

[5] Wyse D.G., Waldo A.L., DiMarco J.P., Domanski M.J., Rosenberg Y., Schron E.B., Kellen J.C., Greene H.L., Mickel M.C., Dalquist J.E. (2002). A comparison of rate control and rhythm control in patients with atrial fibrillation. N. Engl. J. Med..

[6] Kirchhof P., Benussi S., Kotecha D., Ahlsson A., Atar D., Casadei B., Castella M., Diener H.-C., Heidbuchel H., Hendriks J. (2016). 2016 ESC Guidelines for the management of atrial fibrillation developed in collaboration with EACTS. Eur. Heart J..

[7] Camm A.J., Lip G.Y.H., De Caterina R., Savelieva I., Atar D., Hohnloser S.H., Hindricks G., Kirchhof P., Bax J.J., ESC Committee for Practice Guidelines (CPG) (2012). 2012 focused update of the ESC Guidelines for the management of atrial fibrillation. Eur. Heart J..

[8] Boriani G., Cimaglia P., Fantecchi E., Mantovani V., Ziacchi M., Valzania C., Martignani C., Biffi M., Diemberger I. (2015). Non-valvular atrial fibrillation: Potential clinical implications of the heterogeneous definitions used in trials on new oral anticoagulants. J. Cardiovasc. Med..

[9] Sara J.D., Toya T., Taher R., Lerman A., Gersh B., Anavekar N.S. (2020). Asymptomatic Left Ventricle Systolic Dysfunction. Eur. Cardiol. Rev..

[10] Iwagami M., Shinozaki T. (2022). Introduction to Matching in Case-Control and Cohort Studies. Ann. Clin. Epidemiol..

[11] Kwon S., Kim T.J., Choi E.K., Ahn H.J., Lee E., Lee S.R., Ko S.B., Oh S., Lip G.Y.H. (2021). Predictors of ischemic stroke for low-risk patients with atrial fibrillation: A matched case-control study. Heart Rhythm..

[12] Dahlqvist S., Rosengren A., Gudbjornsdottir S., Pivodic A., Wedel H., Kosiborod M., Svensson A.M., Lind M. (2017). Risk of atrial fibrillation in people with type 1 diabetes compared with matched controls from the general population: A prospective case-control study. Lancet Diabetes Endocrinol..

[13] Zaman N., Naccarelli G., Foy A. (2021). A Comparison of Rate Control Agents for the Treatment of Atrial Fibrillation: Follow-Up Investigation of the AFFIRM Study. J. Cardiovasc. Pharmacol. Ther..

[14] Boriani G., Biffi M., Diemberger I., Martignani C., Branzi A. (2003). Rate control in atrial fibrillation: Choice of treatment and assessment of efficacy. Drugs.

[15] Triska J., Tamargo J., Bozkurt B., Elkayam U., Taylor A., Birnbaum Y. (2022). An Updated Review on the Role of Non-dihydropyridine Calcium Channel Blockers and Beta-blockers in Atrial Fibrillation and Acute Decompensated Heart Failure: Evidence and Gaps. Cardiovasc. Drugs Ther..

[16] Horodinschi R.N., Diaconu C.C. (2021). Comorbidities Associated with One-Year Mortality in Patients with Atrial Fibrillation and Heart Failure. Healthcare.

[17] McManus D.D., Rienstra M., Benjamin E.J. (2012). An Update on the Prognosis of Patients With Atrial Fibrillation. Circulation.

[18] Olshansky B., Rosenfeld L.E., Warner A.L., Solomon A.J., O'Neill G., Sharma A., Platia E., Feld G.K., Akiyama T., Brodsky M.A. (2004). The Atrial Fibrillation Follow-up Investigation of Rhythm Management (AFFIRM) study. J. Am. Coll. Cardiol..

[19] Van Gelder I.C., Groenveld H.F., Crijns H.J.G.M., Tuininga Y.S., Tijssen J.G.P., Alings A.M., Hillege H.L., Bergsma-Kadijk J.A., Cornel J.H., Kamp O. (2010). Lenient versus Strict Rate Control in Patients with Atrial Fibrillation. N. Engl. J. Med..

[20] Edoute Y., Nagachandran P., Svirski B., Ben-Ami H. (2000). Cardiovascular adverse drug reaction associated with combined beta-adrenergic and calcium entry-blocking agents. J. Cardiovasc. Pharmacol..

[21] Knight C.J., Fox K.M. (1998). Amlodipine versus diltiazem as additional antianginal treatment to atenolol. Am. J. Cardiol..

[22] Alowais S.A., Hayes B.D., Wilcox S.R., Le J., Koehl J.L., Fuh L. (2021). Heart rate outcomes with concomitant parenteral calcium channel blockers and beta blockers in rapid atrial fibrillation or flutter. Am. J. Emerg. Med..

[23] Schwinger R.H., Böhm M., Erdmann E. (1990). Different negative inotropic activity of Ca^2+^-antagonists in human myocardial tissue. Klin. Wochenschr..

[24] Heywood J.T., Graham B., Marais G.E., Jutzy K.R. (1991). Effects of intravenous diltiazem on rapid atrial fibrillation accompanied by congestive heart failure. Am. J. Cardiol..

[25] Kotecha D., Piccini J.P. (2015). Atrial fibrillation in heart failure: What should we do?. Eur. Heart J..

[26] Goldenberg I.F., Lewis W.R., Dias V.C., Heywood J.T., Pedersen W.R. (1994). Intravenous diltiazem for the treatment of patients with atrial fibrillation or flutter and moderate to severe congestive heart failure. Am. J. Cardiol..

[27] Hirschy R., Ackerbauer K.A., Peksa G.D., O'Donnell E.P., DeMott J.M. (2019). Metoprolol vs. diltiazem in the acute management of atrial fibrillation in patients with heart failure with reduced ejection fraction. Am. J. Emerg. Med..

[28] Multicenter Diltiazem Postinfarction Trial Research Group (1988). The effect of diltiazem on mortality and reinfarction after myocardial infarction. N. Engl. J. Med..

[29] Goldstein R.E., Boccuzzi S.J., Cruess D., Nattel S. (1991). Diltiazem increases late-onset congestive heart failure in postinfarction patients with early reduction in ejection fraction. The Adverse Experience Committee; and the Multicenter Diltiazem Postinfarction Research Group. Circulation.

[30] Kostis J.B., Lacy C.R., Cosgrove N.M., Wilson A.C. (1997). Association of calcium channel blocker use with increased rate of acute myocardial infarction in patients with left ventricular dysfunction. Am. Heart J..

[31] Hager W.D., Davis B.R., Riba A., Moye L.A., Wun C.C., Rouleau J.L., Lamas G.A., Pfeffer M.A. (1998). Absence of a deleterious effect of calcium channel blockers in patients with left ventricular dysfunction after myocardial infarction: The SAVE Study Experience. Am. Heart J..

[32] Figulla H.R., Gietzen F., Zeymer U., Raiber M., Hegselmann J., Soballa R., Hilgers R. (1996). Diltiazem improves cardiac function and exercise capacity in patients with idiopathic dilated cardiomyopathy. Results of the Diltiazem in Dilated Cardiomyopathy Trial. Circulation.

[33] Kotecha D., Holmes J., Krum H., Altman D.G., Manzano L., Cleland J.G.F., Lip G.Y.H., Coats A.J.S., Andersson B., Kirchhof P. (2014). Efficacy of β blockers in patients with heart failure plus atrial fibrillation: An individual-patient data meta-analysis. Lancet.

[34] Trongtorsak A., Kewcharoen J., Saowapa S., Polpichai N., Thangjui S., Navaravong L. (2022). Comparison of mortality rates among rate-control agents in patients with atrial fibrillation: A systematic review and meta-analysis. J. Cardiovasc. Med..

[35] Paolillo S., Dell'Aversana S., Esposito I., Poccia A., Perrone Filardi P. (2021). The use of beta-blockers in patients with heart failure and comorbidities: Doubts, certainties and unsolved issues. Eur. J. Intern. Med..

[36] Bisson A., Ding W.Y., Bodin A., Lip G.Y.H., Fauchier L. (2021). Clinical outcomes with digoxin vs. beta-blocker for heart rate control in permanent atrial fibrillation with heart failure. Eur. J. Heart Fail..

